# A novel unsupervised analysis of electrophysiological signals reveals new sleep substages in mice

**DOI:** 10.1371/journal.pbio.2003663

**Published:** 2018-05-29

**Authors:** Vasiliki-Maria Katsageorgiou, Diego Sona, Matteo Zanotto, Glenda Lassi, Celina Garcia-Garcia, Valter Tucci, Vittorio Murino

**Affiliations:** 1 Pattern Analysis and Computer Vision (PAVIS), Istituto Italiano di Tecnologia, Genova, Italy; 2 Neuroinformatics Lab (NILab), Fondazione Bruno Kessler, Trento, Italy; 3 Genetics and Epigenetics of Behaviour, Neuroscience and Brain Technologies (NBT), Istituto Italiano di Tecnologia, Genova, Italy; 4 Department of Computer Science, University of Verona, Verona, Italy; UCSF School of Medicine, United States of America

## Abstract

Sleep science is entering a new era, thanks to new data-driven analysis approaches that, combined with mouse gene–editing technologies, show a promise in functional genomics and translational research. However, the investigation of sleep is time consuming and not suitable for large-scale phenotypic datasets, mainly due to the need for subjective manual annotations of electrophysiological states. Moreover, the heterogeneous nature of sleep, with all its physiological aspects, is not fully accounted for by the current system of sleep stage classification. In this study, we present a new data-driven analysis approach offering a plethora of novel features for the characterization of sleep. This novel approach allowed for identifying several substages of sleep that were hidden to standard analysis. For each of these substages, we report an independent set of homeostatic responses following sleep deprivation. By using our new substages classification, we have identified novel differences among various genetic backgrounds. Moreover, in a specific experiment with the *Zfhx3* mouse line, a recent circadian mutant expressing both shortening of the circadian period and abnormal sleep architecture, we identified specific sleep states that account for genotypic differences at specific times of the day. These results add a further level of interaction between circadian clock and sleep homeostasis and indicate that dissecting sleep in multiple states is physiologically relevant and can lead to the discovery of new links between sleep phenotypes and genetic determinants. Therefore, our approach has the potential to significantly enhance the understanding of sleep physiology through the study of single mutations. Moreover, this study paves the way to systematic high-throughput analyses of sleep.

## Author summary

Sleep is a heterogeneous process determined by a number of genetic and epigenetic factors. To investigate the biology of sleep, animal models, such as mice, are extensively used in sleep studies, and large-scale phenotypic datasets are required to reach meaningful conclusions. Currently, manual annotations of electrophysiological states by a researcher is the gold-standard approach to classifying sleep stages. Only a few sleep states are identified through such manual annotations, namely non-rapid-eye-movement (NREM) and rapid-eye-movement (REM) sleep. In this work, we present a new computational approach that identified multiple new substages of sleep based on standard electroencephalography (EEG)/electromyography (EMG) features. Using this new approach, we studied each individual identified state and discovered that many of these states respond to the basic principles of sleep physiology: for example, some states homeostatically respond to sleep deprivation. We also applied our method to different mouse strains and to a circadian mutant line of mice. In all experimental groups, we were able to refine our understanding by associating specific substages with the genetic variations. We conclude that our new unbiased computational approach can help refine the study of sleep by further dissecting sleep biology.

## Introduction

Sleep is a physiological, metabolic, and behavioral state of the organism that plays an important role in many biological functions. It is described by 2 different states—namely, non-rapid-eye-movement (NREM) sleep and rapid-eye-movement (REM) sleep. In laboratory settings, sleep is conventionally defined by properties of electrophysiological signals, and many features of these biological signals are shared across species. For this reason and thanks to the advances in mouse genetics, the study of sleep in mice has become popular in many laboratories.

A guideline for sleep scoring was developed for human studies as a tool for visual analysis of electroencephalography (EEG) and electromyography (EMG) traces according to a predefined set of rules [1]. Based on these rules, a simplified catalog of electrophysiological properties was determined for rodents as well. Differently from humans, in rodents NREM sleep is considered to be a single state. It is characterized by low EEG frequencies (below 5 Hz) with a progressive reduction of muscular tone, and it is usually referred to as deep sleep. On the other hand, REM sleep is characterized by EEG frequencies in the range 5–9 Hz. This makes it an electrophysiologically active state, similar to wakefulness in many aspects, even though the muscle tone decreases dramatically during this state. Due to this fact, REM sleep is also called paradoxical sleep. These basic criteria and some additional properties (i.e., periodic electrophysiological phenomena that occur in EEG) are used to manually classify long series of time epochs (each epoch usually lasts 4 seconds) into either one of the two aforementioned sleep states or into the wakefulness state.

Visual scoring of the different time epochs is the current gold-standard approach that provides fundamental insights into sleep regulation and physiology; however, it is a time-consuming process that can be affected by errors and misjudgments across different scorers. This is caused by the subjective decisions that can easily bias the labeling process. Indeed, inter- and intrascorer variability is always present, and studies evaluating the consistency between scorers have shown an average agreement of about 80%, which varies significantly across different sleep states [2,3,4]. In addition to the difficulties related to manual scoring, it is well recognized that sleep states are nonhomogeneous units representing an aggregation of different substages [5]. For example, NREM sleep in humans is typically characterized by 3 substages, and in some animals, it can be divided into 2 substages (slow-wave sleep I and II) [6]. However, whether sleep stages should be divided into multiple substages, each one having a physiological relevance, remains an open question in sleep biology.

In this scenario, we attempted to go beyond the current state of knowledge in the study of sleep in mice, trying to extrapolate unforeseen phenomena from the data. Our study is driven by the assumption that a richer structure of electrophysiologically derived properties can be discovered from standard datasets. Thus, we present here a different conceptual and technical framework for the analysis of sleep in mice based on the adoption of unsupervised machine learning. The proposed scheme has the potential to represent a paradigm shift in the study of sleep, allowing for an investigation without prior assumptions on the different states, thus facilitating an agnostic exploratory data analysis. Indeed, employing models that are capable of detecting regularities in large datasets, we can discover many sleep substages that a human scorer would never detect by a standard visual inspection of signals.

**Fig 1:**
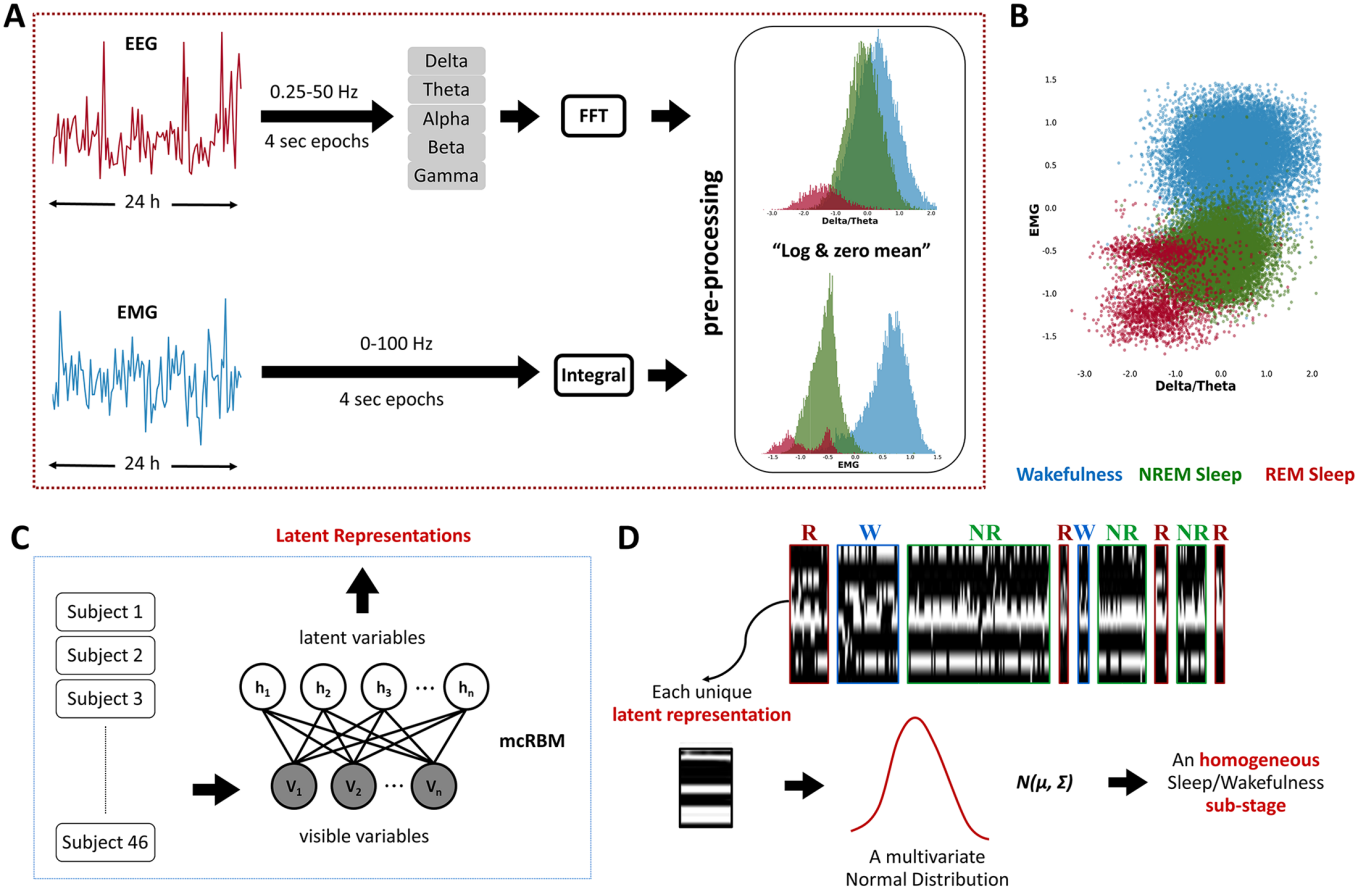
Multisubject data analysis pipeline. (A) Data collection and preprocessing per subject; FFT was applied to EEG signals to derive the power histogram of 5 bands (Alpha, Beta, Delta, Gamma, and Theta), and the ratios between all possible pairwise combinations of the 5 EEG bands were computed. For the EMG, we used its integral values. Each individual dataset was preprocessed by taking the natural logarithm of the data and zeroing the mean of each feature separately. (B) Scatter plot of the log-(Delta/Theta) and the log-EMG features showing the existence of more clusters in the samples of one subject. Notice that REM sleep is characterized by at least 2 substages. Data points belong to a single-subject time series. Blue, green, and red samples are associated with wakefulness, NREM sleep, and REM sleep, respectively, as hand-labeled by the expert. (C) Data modeling: time series of EEG/EMG data of all subjects were concatenated into a unique dataset and modeled with an mcRBM, resulting in a set of latent variables’ representation of the input data. (D) Evolution of mcRBM’s latent variables activity while processing an input time series in which, for a visualization purpose, the sequence of states has been grouped according to the 3 known stages, i.e., NREM (NR), REM (R), and wakefulness (W), as defined by the manual scoring. Time flows from left to right, with each pixel-wide column representing 1 time step (4 s epoch) and each pixel-wide row representing 1 latent variable. Black pixels represent nonactive units, while white pixels represent active ones. It is interesting to see how the model mostly gives similar configurations to epochs that belong to the same sleep stage. For this reason, blocks that belong to the same stage look similar to each other. Notice that units that are active in REM are generally not active in NREM and wakefulness (units in yellow frame). With the mcRBM, we are fitting Gaussian distributions to the data. Hence, each single binary representation is associated with a multivariate normal distribution [8]. EEG, electroencephalography; EMG, electromyography; FFT, fast Fourier transformation; mcRBM, mean-covariance restricted Boltzmann machine; NREM, non-rapid eye movement; REM, rapid eye movement.

The class of latent (hidden) variable models have proven to be very effective in capturing hidden regularities in the data, due to their ability to encode rich structured priors and infer latent properties of the data without requiring human annotations [7]. Out of all potential models, we decided to adopt the mean-covariance restricted Boltzmann machine (mcRBM) [8] to model brain and muscle activities. This model is capable of learning the joint distribution between a set of continuous observed random variables and a set of binary unobserved (latent) ones, modeling complex (multimodal) distributions from Gaussian-like data (like in our case, see Fig 1A), and exploiting correlations between the input variables. Specifically, it allows inferring a set of latent representations (namely, latent states) describing different modes in the input data distribution, each one ideally corresponding to a cluster associated with a reoccurring sleep pattern. These latent states can be therefore interpreted as model descriptors identifying different sleep substages. For this reason, in the following text, we will interchangeably refer to them as latent states or substages.

This model has been demonstrated to be capable of learning such regularities and discovering meaningful phenomena [7]. Indeed, thanks to the unsupervised nature of the approach, we were able to study unforeseen complex phenomena appearing in electrophysiological data that cannot be studied with common rule-based and supervised learning approaches [9,10,11,12] just because such phenomena are not entirely modeled or described by the experts’ prior knowledge. This approach, for the first time, allowed the identification of multiple substages both in sleep and wakefulness phases. Thus, we explored the variability in the sleep behavior between different mouse genotypes. In particular, we performed a multisubject analysis applied on groups of mice with different genetic backgrounds, i.e., a classical pure inbred mouse strain, an outbred strain, and a strain with mixed background. From this multisubject data analysis, we identified a repertoire of novel substages that characterize the different physiological states of mouse sleep. Taking this as a starting point, we identified novel sleep-behavioral differences among mouse genotypes. Indeed, the results of our experiments robustly show that there are several substages that are characteristic of 1 strain, and this can be informative of the genetic differences across mice.

In an effort to further investigate the use of our approach, we applied our analysis to the recently identified *Zfhx3*^*Sci/+*^ mouse strain, which presents a shortening of the circadian clock and alterations in sleep homeostasis [13]. Our new approach provides a deeper understanding of the sleep abnormalities in this circadian mutant mouse line, revealing specific EEG anomalies and circadian modulations. We assessed the physiological relevance of each new stage by testing the homeostatic response to a perturbation (i.e., sleep deprivation). Thus, we identified a general heterogeneous homeostatic response, suggesting that these states carry a potentially unique physiological role. Yet we observed that specific substages show distorted responses in mutants’ sleep, which may indicate the presence of subtle microstructures in the sleep of mice that are governed by single genes.

It is important to highlight that, beyond being a novel approach to the analysis of complex sleep phenomena, the proposed method allows management of sleep longitudinal studies, transferring the inferred knowledge across experiments. In addition, it can also be applied to other investigations in any biological domain involving the analysis of similar data (i.e., time series), such as other behavioral studies.

## Results

### Identification of new sleep–wakefulness substages

We explored the homogeneity of the 3 standard physiological states (NREM sleep, REM sleep, and wakefulness) using a cohort of 46 animals from 3 different strains: (i) a common inbred mouse strain (C57BL/6J); (ii) a mixed background strain (BALB/c × C3H/HeH × C57BL/6J); and (iii) an outbred mouse line (CD1). We investigated the existence of substages that are common across animals through a multisubject data analysis pipeline. To deal with the subject-to-subject variability, each individual dataset was preprocessed separately, obtaining a uniform representation across subjects (see Fig 1A). After preprocessing, it was possible to observe the presence of nonhomogeneous state distributions. For example, Fig 1B shows in one animal that REM sleep episodes are distributed across at least 2 main clusters.

**Fig 2:**
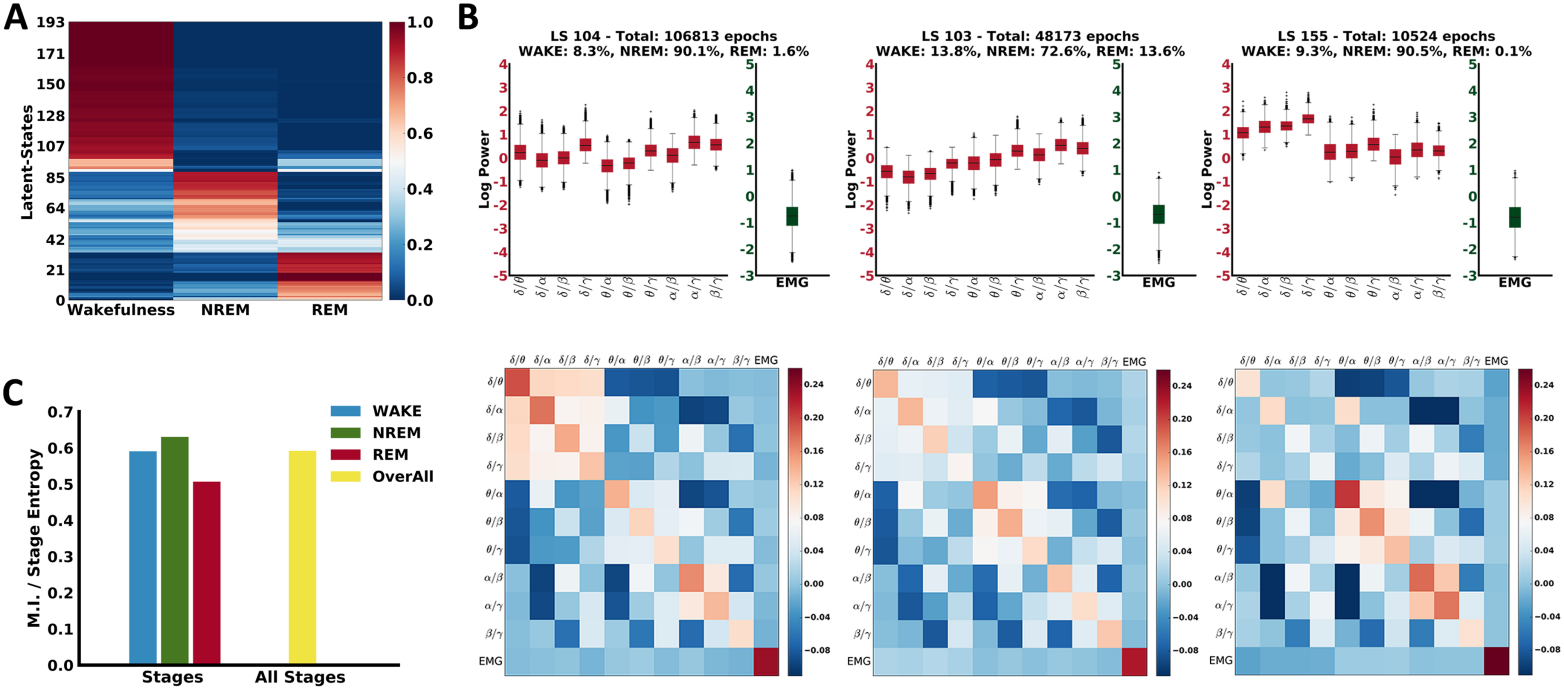
Evaluation of the observed substages. (A) Probability matrix of LS being associated with the known sleep stages. Rows refer to LS and columns to wakefulness, NREM, and REM sleep stages. Color shades range from blue, representing low probability, to red, representing high probability, for the corresponding LS to belong to each of the 3 known stages. The order of the substages in the graph has been achieved using agglomerative hierarchical clustering. (B) Analysis of the NMI between the observed substages and the 3 stages. Each bar represents the NMI per stage. The right bar corresponds to ratio assuming as 2 random variables the “substages” and the “three stages.” (C) Examples of distributions of the input features associated with 3 substages corresponding with high probability to the NREM stage. The top row shows the distributions of the input samples in a box plot. The bottom row shows the covariance between the input variables. EMG, electromyography; LS, latent states; MI, mutual information; NMI, normalized mutual information; NREM, non-rapid eye movement; REM, rapid eye movement.

Since we were looking for co-occurring regularities in the data, aiming at discovering common sleep substages across subjects and across groups, all individual datasets of the aforementioned 3 mouse lines were modeled jointly with a single mcRBM (Fig 1C). This allowed us to search for regularities within the dataset, inferred through the states of the model’s latent variables (Fig 1D). As a result, approximately 190 different latent states (i.e., binary configurations of latent variables) were identified, most of which were strongly representative of the labeling performed by manual scoring. In Fig 2A, we provide a visual representation of this relationship, showing the probability for each latent state (rows) to correspond to each of the 3 manually scored sleep states (columns). From this probability matrix, we can observe that a significant number of latent states map with high probability to only 1 of the 3 known states. There are also some latent states that could be associated with approximately equal probability (whitish color in the graph) to at least 2 states. For example, there are latent states mapping to both wakefulness and REM and others mapping to both NREM and REM. While these latter latent states are likely to be misclassified in the classical scoring system—for example, by different scorers—in our unbiased classification, they are independent states with a specific frequency and timing.

We also measured how informative the different latent states are about the 3 states by estimating the normalized mutual information (NMI) [14], which measures how well the learnt latent states have encoded the sleep states. In our case, it was approximately 0.6, indicating a good informativeness of the latent states. Mutual information (MI) and state entropy were also computed for each state separately and, as shown in the graph in Fig 2B, the observed latent states are highly informative about the 3 states (i.e., NMI > 0.5).

Thanks to the fact that the mcRBM is a generative model, we were able to further investigate the variability of the input data distributions associated with each inferred latent state. Specifically, a multivariate normal distribution (represented by its mean vector and covariance matrix) over the input variables was inferred for each latent state (see Fig 1D). Different latent states correspond to different distributions in the input data, even when they can be statistically associated with the same sleep stage (see Fig 2C and S1 Fig). This result points to the existence of a more elaborate electrophysiological characterization of sleep than expected, showing that the 3 main states are nonhomogeneous entities. This supports our hypothesis that the known 3 sleep stages can be described by multiple latent states in the model, hence by different sleep substages that experts cannot detect by a simple visual inspection of the signals.

### Substages discriminate mouse genotypes

**Fig 3:**
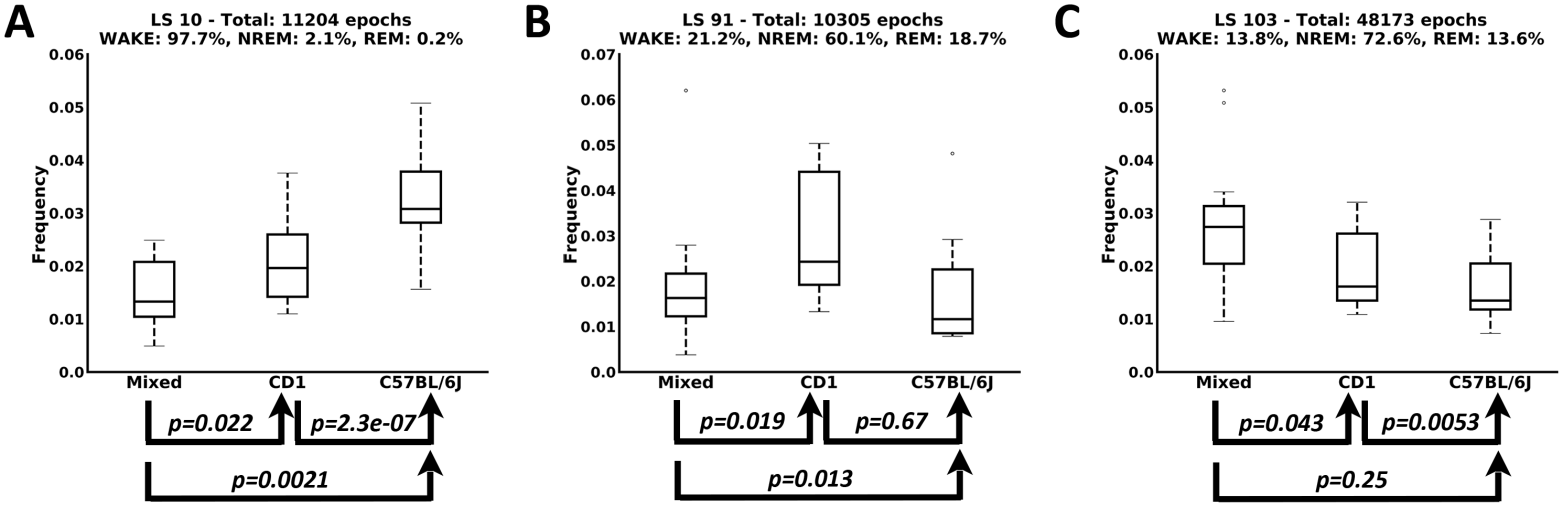
Examples of 3 substages that are characteristic of the 3 mouse genetic backgrounds. Box plots describing the statistics on the number of epochs each strain falls in the corresponding substage. Arrows below each graph show the *p*-values for the 2-sample independent *t* test computed on the top distributions. (A) A substage associated with the wakefulness stage for which the C57BL/6J mouse group is significantly different from the other 2 groups (*p*-values < 0.05). (B) A substage mapping to the NREM stage that is characteristic for the CD1 strain. (C) A substage associated with NREM sleep that is characteristic for the mixed background group. LS, latent stage; NREM, non-rapid eye movement; REM, rapid eye movement.

In light of the findings described in the previous section, we investigated whether there are substages that are characteristic of specific mouse genotypes. Indeed, we observed that a significant number of substages are mostly associated to 1 of the 3 strains. In order to determine those that best describe each strain, we used the 2-sample independent *t* test, comparing the distributions of the substages across the mouse genotypes. Three examples are shown in Fig 3, in which the graphs depict for each strain the statistics on the number of epochs in which each latent state is observed. The arrows below each graph show the *p*-values of the 2-sample independent *t* test, giving a picture of the difference between each combination of 2 distributions. This approach allows determining analytically the sets of all latent states that best characterize each single genotype.

Prompted by the variability of sleep among mouse groups, we attempted to test whether we can use the substages to discriminate between the mouse genetic backgrounds with a supervised machine-learning approach. Each group and each subject is described by a discrete probability distribution over the observed latent states. In a leave-one-subject-out classification framework and using a majority-voting criterion, we achieved a discrimination performance of 85.11%. The resulting confusion matrix is shown in S2 Fig, in which we can see that only 2 subjects for each strain have been misclassified. We can also observe that both misclassified subjects of the mixed background strain have been classified as belonging to the C57BL/6J group, which is interesting because the C57BL/6J background and mixed background groups are genetically related. This experiment highlights the power of our study in detecting genetic background effects. Since different genetic backgrounds are associated with specific traits, our capability to discriminate between mouse groups indicates that some latent states we identified depend on specific genetic components.

### Transitions across substages show a robust clustering of sleep and wakefulness

**Fig 4:**
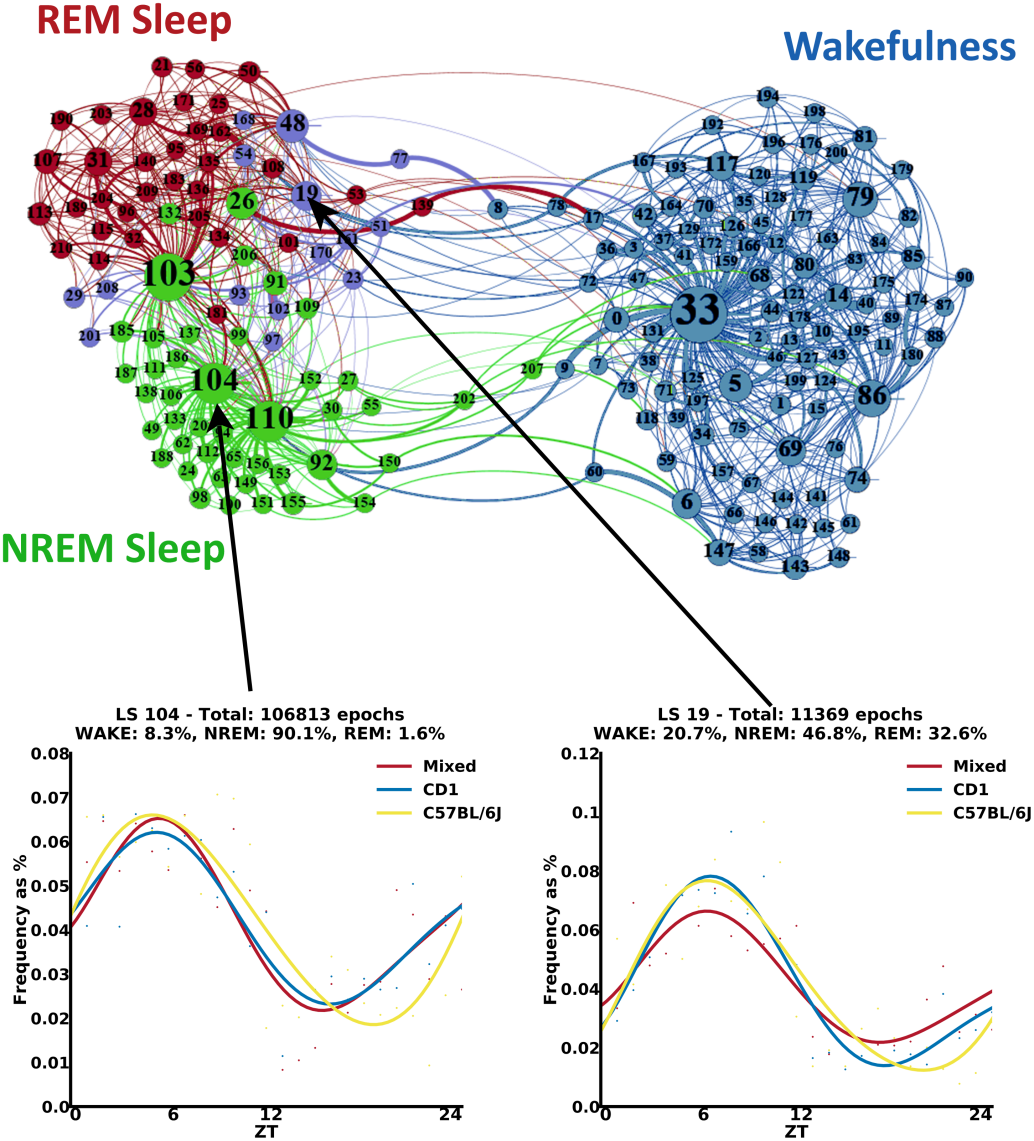
Transitions’ probabilities between substages and daily profiles analysis. Top: state-transition graph in which nodes have been clustered according to the transition probabilities. Nodes correspond to the substages, and edges identify the transitions between stages. Nodes’ size is related to their in-degree. Blue, green, and red nodes are associated with substages mapping with high probability to wakefulness, NREM sleep, and REM sleep, respectively. Purple nodes correspond to substages that do not have a clear mapping to 1 of the 3 known stages (probability lower than 0.6 for any stage). Edges’ weight is related to the corresponding transition probability, and their color is related to the source node. Graphs were built using the ForceAtlas2 algorithm [15,16] (see also interactive graphs at http://pavis.iit.it/datasets/mouse-sleep-analysis). Bottom: examples of daily profiles associated with NREM sleep, in which the profile of C57BL/6J mouse group is slightly shifted. Histograms describe the distribution of epochs using bins of 1 hour. Polynomial curves are fitted to the histograms to obtain a simplified visual representation of daily oscillations. LS, latent state; NREM, non-rapid eye movement; REM, rapid eye movement; ZT, zeitgeber time.

Transitions between different substages can be informative in the analysis of sleep over 24 hours. We computed the transition probabilities between the latent states for the entire dataset. As expected, latent states mapping to a certain sleep stage among NREM, REM, and wakefulness have higher probability to be followed by latent states mapping to the same stage. We visualized the transition probabilities in a graph where nodes (the latent states) are clustered according to their overall connectivity using a multidimensional scaling algorithm (Fig 4 top). The absolute position of each node in this graph is not relevant; the overall grouping of nodes is what can give us useful insight. Indeed, as a first result, we noticed that clustering the latent states according to their connectivity surprisingly also results in a meaningful grouping of nodes in terms of the 3 known sleep stages. The clustered graph confirms basic knowledge regarding the transitions across sleep states. For example, the cluster of latent states mapping to NREM sleep is near to the cluster of latent states mapping to REM sleep, and both are far away from the cluster of latent states associated with wakefulness. Interestingly, latent states not having a clear mapping to any of the 3 known sleep stages (whitish substages in Fig 2A) are usually interleaving substages positioned between the 3 overall clusters and associated with a transitional state between the 3 known sleep stages (see purple nodes in Fig 4).

In a deeper investigation of the differences among mouse genetic backgrounds, we also built the graphs for each strain separately, following the same principle (see graphs in S3 Fig and interactive graphs at http://pavis.iit.it/datasets/mouse-sleep-analysis). This allowed us to look for possible differences among the 3 groups. We searched for nodes assuming a different role in the 3 strains (e.g., nodes that are central to a cluster in a group and become transitional between 2 clusters in another graph). Indeed, even though the general clustering is common across graphs, there are some nodes whose role changes significantly. For instance, node 53, while being associated with REM sleep according to human labeling, seems to be a transitional state between wakefulness and NREM sleep in the graph generated from the CD1 strain. On the contrary, the same node in the graphs generated from both C57BL/6J and mixed background groups has transitions only with NREM and REM sleep nodes (see black arrows pointing out this node in each graph in S3 Fig). Similarly, node 174 in the mixed background group appears as a transitional state between wakefulness and sleep, while in the other groups, it has transitions only with nodes associated with wakefulness (see orange arrows pointing out this node in each graph in S3 Fig). These results imply that specific substages can have different roles according to a specific genetic background and cannot be considered as isolated stages. This offers an unprecedented dynamic aspect in studying sleep physiology within multiple sleep microstructures.

### Substages follow daily variations

To investigate how substages develop over time, we analyzed the daily behavior over the 24 hours for each genotype. Specifically, we computed the histogram of the 4-second epochs that fall in each single latent state using bins of 1 hour, starting from 7:00 AM. Histograms are computed for each latent state and for each group separately. A polynomial curve is fitted to the histogram to obtain a representation of daily oscillations. We observed that some latent states show different daily profiles across mouse genotypes. For example, the profile of the C57BL/6J strain in latent state 104 (see Fig 4 bottom left) is shifted compared to the other 2 strains. Interestingly, the latter state (as well as latent state 110 in S4B Fig) is quite numerous within the dataset and particularly in NREM sleep, suggesting that the daily distribution of the latent states is an additional powerful discriminator of mouse genotypes. A similar shift in the profile of C57BL/6J was observed in many other latent states, including those that do not have a clear mapping to only 1 of the 3 known sleep stages, like latent state 19 (see Fig 4 bottom right). In S4 and S5 Figs, we report further examples of daily profiles of specific latent states and the manually annotated known sleep stages, respectively. This shift in the profile of C57BL/6J is present in almost all the latent states, as well in the 3 known stages, and it is due to its genetic background that, to the best of our knowledge, it has not been described in literature before.

### During the subjective night, latent states are divided in early and late peaks

**Fig 5:**
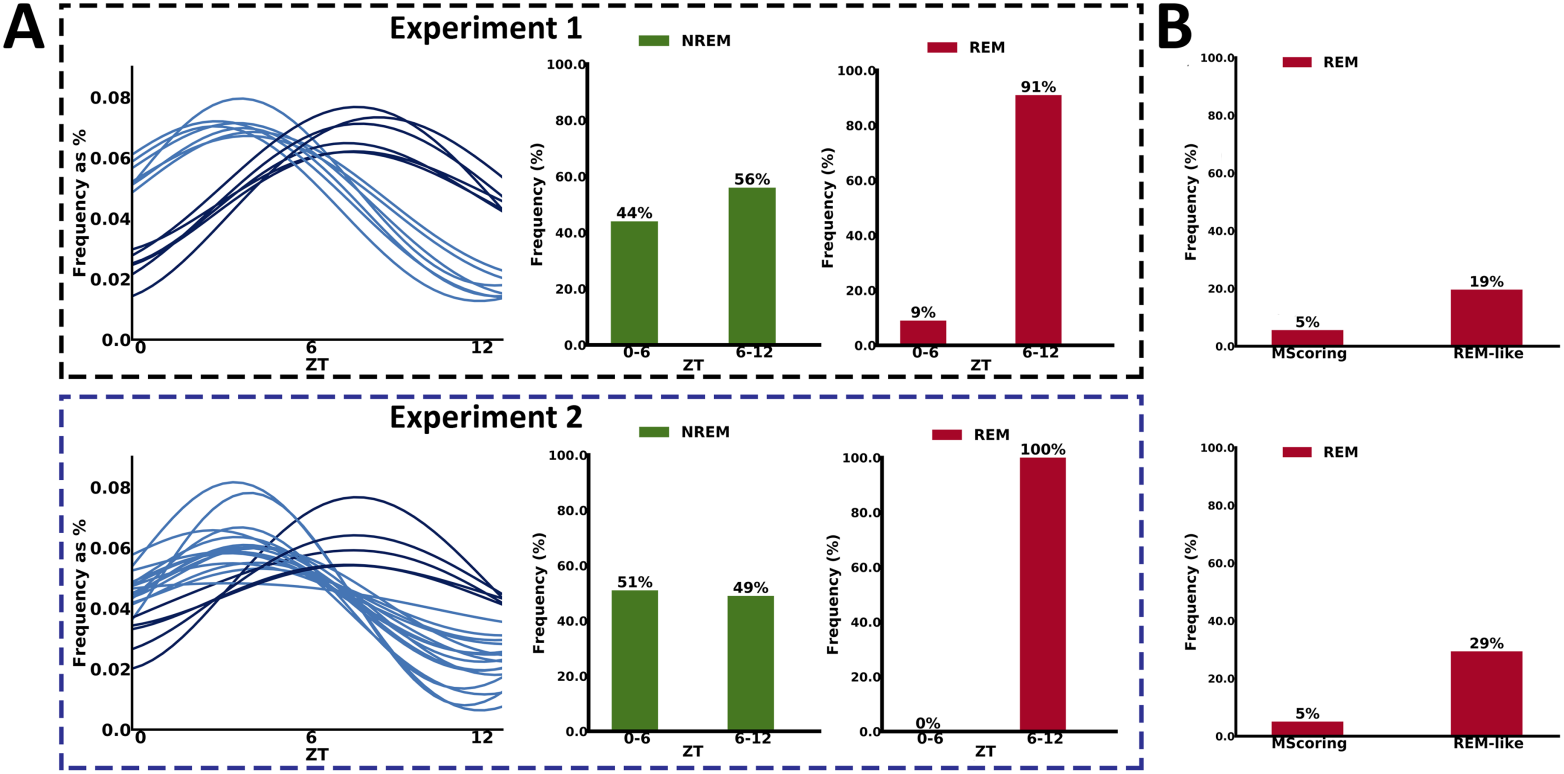
Temporal profile of REM-like states. (A) Analysis of the peaks during the light phase (07:00–19:00) identified 2 populations of latent states, presenting peaks either in the first or the second half of the subjective night. Top graphs are associated with the first experiment (data coming from the 3 wild-type genetic backgrounds). Bottom graphs are associated with the second experiment (*Zfhx3*^*Sci/+*^ mutant strain versus *Zfhx3*^*+/+*^ littermate controls). In both experiments, the majority of the substages that map into the second half of the light phase has a high probability to be classified as REM (therefore REM-like). The substages classified as NREMs are equally divided into the first and second half of the light phase. (B) Comparison between manually annotated REMs versus REM-like states generated by our approach, which peak in the second half of the light phase, in the 2 experiments. Graph shows the percentage of epochs labeled as REM (refer also to S1 Data). NREM, non-rapid eye movement; REM, rapid eye movement; ZT, zeitgeber time.

We further analyzed the latent states having maximum peaks during the subjective night (light phase: 7:00–19:00). We observed that there is a group of latent states having maximum peak in the first half of the light phase, while some others have a peak in the second half of the light phase. Interestingly, we observed that 91% of the latent states mapping with high probability to REM have peaks in the second half of the light phase (see Fig 5A). This compartmentalization of REM-like sleep substages across the subjective night resembles the classical distribution of REM sleep in humans, in which the majority of REM sleep occurs in the second part of the night. Our result, suggesting that a microstructure of REM-like sleep occurs in the second part of the subjective night of a mouse, was never described before. Thus, we can speculate that the temporal profile of these latent states could be informative of the full REM-like sleep in mice. Indeed, REM sleep is often reported to be 5%–10% of sleep in mice, while in humans, it reaches up to 20%. In Fig 5B, we compared the percentage of REM sleep annotated with manual scoring versus the percentage of REM-like sleep, considering all latent states that present a profile that peaks in the second half of the subjective night. Remarkably, the account of latent states is close to the percentage (in range of 20%–30%, see Fig 5B) of REM sleep reported in humans. These results may suggest that this new method has the potential to describe a microstructure of REM-like sleep states in mice that resemble the human one.

### The latent states are homeostatically regulated and genetically determined

In order to explore in more detail the idea of using specific latent states as biomarkers for the identification of single gene determinants (i.e., in circadian clock alterations), we also performed an additional sleep analysis of the *Zfhx3*^*Sci/+*^ mutant strain, which is characterized by a reduced circadian period and some other differences in their sleep architecture compared to their littermate control *Zfhx3*^*+/+*^ mice [13].

**Fig 6:**
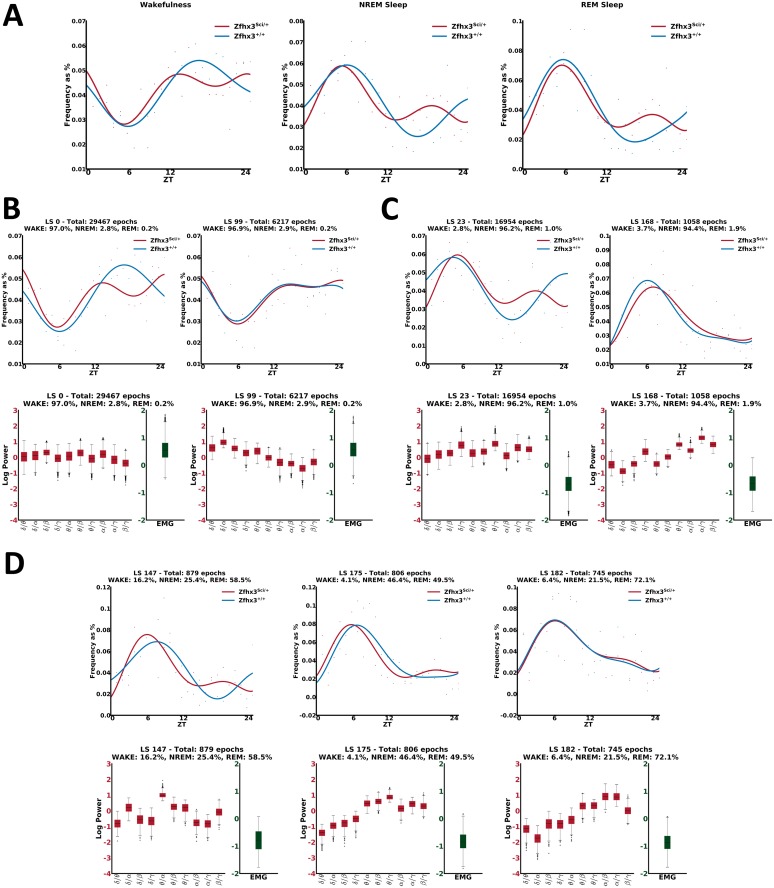
Daily behavior analysis in the circadian mutants. (A) Profiles per sleep stage of Zfhx3^Sci/+^ versus Zfhx3^+/+^, using the manually scored epochs. (B) Examples of substages mapping with high probability to wakefulness. Left: the Zfhx3^Sci/+^ mutants show a reduced profile compared to the Zfhx3^+/+^. Right: the profiles of both groups are flat during the dark phase. As can be observed from the graphs on the bottom, also the distributions of the input variables across the 2 substages are different. (C) Examples of substages mapping with high probability to NREM. Similarly to C, there are substages where the Zfhx3^Sci/+^ mutants show a reduced profile compared to the Zfhx3^+/+^ (left), as well as substages where the profiles of both groups are flat during the dark phase (right). Also, in this case, the distributions of the input variables across the 2 substages are different (bottom graphs). (D) Top left and middle graphs: examples of substages mapping with high probability to REM, in which we observed that the profile of Zfhx3^Sci/+^ has an advance compared to the one of Zfhx3^+/+^. Top right: example of a substage in which no difference is observed across the 2 groups. Bottom: Differences in the input data distributions can be observed across the 3 examples. EMG, electromyography; LS, latent state; NREM, non-rapid eye movement; REM, rapid eye movement; ZT, zeitgeber time.

Following the same processing pipeline as in the previous experiment, all datasets of the 2 groups were modeled jointly with an mcRBM. Analyzing the daily profile of the inferred configurations, we observed the existence of latent states where *Zfhx3*^*Sci/+*^ mutants have a reduced profile (see Fig 6B and 6C left). This is also evident from the analysis of the profiles of the 3 known states using the manually scored epochs (see Fig 6A), as was already shown in [13]. Moreover, some latent states show flat profiles during the dark phase for both strains (see Fig 6B right and Fig 6C right), showing more specific information in the data that is not evident when only relying on standard scoring. Looking at the mean of the distributions over the input data (see bottom plots in Fig 6B and 6C), we also observed that such states have different data distributions compared to the previous cases. Finally, we also noted that for some latent states, mainly associated with REM or NREM stages, the profile of *Zfhx3*^*Sci/+*^ mutants has a forward shift compared to their littermate control group (see Fig 6D, first 2 top graphs).

**Fig 7:**
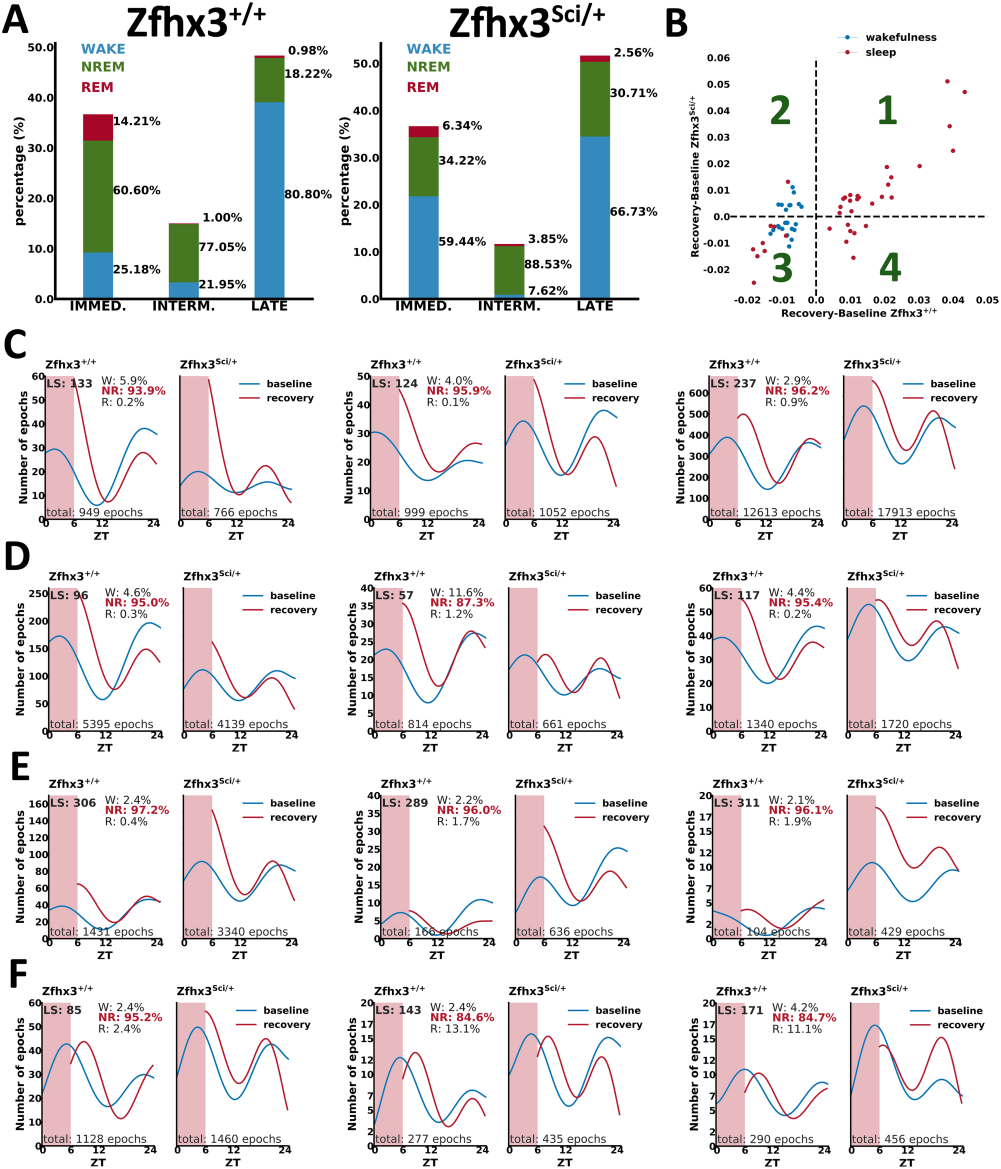
Homeostatic responses of latent states. (A) Percentage of peak rebounds at ZT 6 (immediate), within ZT 6 and ZT 12 (intermediate), and beyond ZT 12 (late). Within each category, the distribution of wake (blue), NREM (green), and REM (red) stages according to the manual annotation is represented (refer also to S2 Data). (B) Analysis of responses in Zfhx3 mutants versus wild-type mice. Each dot describes the difference between the rebound and the baseline phase of a latent state for the same period of the day. Examples of latent-state occurrences are presented for states presenting no differences between mutants and wild-types (C); for states in which mutants present reduced response (D); for states in which mutants present higher response compared to wild-types (E); and for states in which both groups are not responding, then not homeostatically regulated (F). Note that the shaded region in the plots C-F corresponds to the period of sleep deprivation. NREM, non-rapid eye movement; REM, rapid eye movement; ZT, zeitgeber time.

A further validation of the physiological value of each latent state came from the study of how each substage responds to sleep deprivation. Indeed, sleep is a homeostatic process that is best described by its rebound response following sleep deprivation; hence, we tested it for each latent state. In Fig 7A, we show the distribution of rebound responses in Zfhx3 wild-type and mutant mice. In particular, we classified between immediate (i.e., at zeitgeber time [ZT] 6), intermediate (i.e., within ZT 6 and ZT 12), and late (i.e., beyond ZT 12) responses over the 6 hours following sleep deprivation. The dynamic of rebound responses in the 2 groups of mice is similar, with both groups having similar peaks at different times along the recovery phase (Fig 7A). Then, we looked at the rebound for each latent state by assessing the different response compared to its baseline value for the same time of the day (Fig 7B shows the amount of rebound of latent states for the 2 models). This analysis provides an initial screening of latent states that present similar responses in the 2 groups (i.e., linearly correlated in Fig 7B corresponding to quadrants 1 and 3) and latent states that respond differently according to the genotype (i.e., nonlinearly correlated in Fig 7B corresponding to quadrants 2 and 4). Thus, we performed a more detailed analysis of individual responses to sleep deprivation, and this provided a set of heterogeneous behaviors across different latent states, ranging from high-frequency states (e.g., >17,000 epochs) to low-frequency ones (e.g., <1,000 epochs). As we can appreciate from Fig 7C, some latent states respond equally in the 2 groups of mice. The analysis also revealed specific latent states in which the homeostatic rebound following sleep deprivation is attenuated or fully suppressed in mutants compared to wild-type mice (Fig 7D). We also identified states in which the response to deprivation is increased in mutants (Fig 7E) or not present in both groups (Fig 7F). Overall, the results of applying our approach to a perturbed condition such as sleep deprivation indicate that the latent states we have identified are independent states that can differentially respond to homeostatic rebound and that single gene change may influence each state separately.

## Discussion

Our study presents an advancement in the methodologies allowing the study of EEG-based sleep physiology in mice. Using our approach, we were able to identify sleep substages using standard electrophysiological data collected from mice. The observed sleep substages are well contained within the standard NREM sleep, REM sleep, and wakefulness categories, providing a preliminary validation of the proposed approach. Moreover, this new unbiased method also provides a rich repertoire of substages in wakefulness, which are, however, difficult to characterize because they correspond to a large range of behaviors. Therefore, we focused our study mainly on the latent states that emerge within sleep. Our method, by its own nature, can be used independently from classical NREM/REM annotation, offering an unbiased approach to the analysis of sleep states.

In order to validate the biological relevance of the learnt latent states, we applied our method to a multisubject analysis framework including different groups of mice. Specifically, we used 3 groups of mice representing the most commonly used genetic backgrounds in mouse genetics. Our study has shown that a number of latent states are characteristic of specific genotypes, and this greatly enriches the study of the genetic components of EEG sleep. Indeed, the results of our study advocate for a new set of endophenotypes that can be studied in mice by reanalyzing electrophysiological data. Interestingly, we also observed that the majority of the latent states follow a circadian-like profile and are homeostatically regulated, which adds further information in the physiological relevance of states determined by our approach. Furthermore, the definition of latent states according to the circadian phase in which they are expressed or according to their homeostatic response after sleep deprivation generates new scientific questions to be investigated. For example, our study on the *Zfhx3* circadian mutants refined the understanding of the sleep patterns affected by the mutation. In particular, we identified specific states that can account for increased sleep episodes in the dark phase of mutants. Within the NREM alterations reported for this line, we were also able to identify specific latent states that are characteristic of the mutants. Moreover, the oscillation of specific mutant states over 24 hours is shortened compared to littermate control mice, suggesting a specific correlation between sleep physiology and circadian clock. Yet, reanalyzing the homeostatic defect of this mutant line by means of the proposed analysis framework, we noticed that some specific latent states can account for the genotype differences, while this is not possible considering the whole NREM stage. Moreover, the mutation may be characterized by various latent states presenting either reduced rebound or increased rebound; therefore, the overall effect we observe in the mutants is the result of a highly dynamic sleep structure. This latter example indicates that our approach may be applied to additional datasets of sleep and circadian mutants. The general outcome emerging from our study is that, as expected, sleep in mice is more complex than what was analyzed so far, and applying our multistage dissection of sleep may highlight different dynamic structures of sleep that are physiologically relevant and genetically determined.

In the future, our methodological pipeline can be applied to datasets coming from different mouse mutants, promising a deeper understanding of the influence of genetic variations on specific physiological processes in sleep.

Last but not least, our method can naturally cope with a large dataset, allowing it to be employed in the analysis of high-throughput experiments, which are very much needed in large-scale screening in mouse genetics and drug discovery. Indeed, despite its unsupervised nature, the presented analysis pipeline can easily be turned into an automatic tool for scoring sleep in mice. Moreover, although it was out of the scope of the current work, the proposed approach allows the study of the physiological role of specific latent states corresponding to interesting modes, i.e., multivariate normal distributions over the input variables. In principle, it is possible to label relevant modes, creating a set of ground-truth distributions that can be used to identify and label the substages in new experiments.

## Methods

### Ethics statement

All animal procedures were approved by our institutional animal committee (“*Organismo preposto al benessere degli animali*”, OPBA, IIT, Genova) and by the ethical national committee in Italy for IIT Genova. All procedures were done under the Italian Policy (license issued on 19 June 2009, decreto N^o^106/2009-B). The study followed ARRIVE guidelines (http://www.nc3rs.org.uk/arrive-guidelines, see S1 Checklist). All mice were anesthetized IP with Ketamine/Xilazine, 90-150K/7.5-16X and implanted with telemetry transmitters (Data Sciences, F20-EET, Gold system) for recording EEG.

### Animals

We analyzed EEG/EMG recordings coming from 46 male mice of 3 different groups:

- a classical inbred strain: C57BL/6J (*n* = 13 subjects),
- an outbred strain: CD1 (*n* = 14 subjects),
- and a mixed background group (BALB/c × C3H/HeH × C57BL/6J; *n* = 19 subjects).

Isogenic C57BL/6J mice are the most widely used mice in neuroscience due to the fact that, as with any other inbred genetic background model, it reduces variability in phenotypic expression. Moreover, a number of mouse mutant models have been developed and are available on C57BL/6J background. However, in the study of mouse models for translational medicine, the testing of mutations on mixed background models (e.g., BALB/c × C3H/HeH × C57BL/6J) or outbred mice (CD1), which resemble human genetic variations better, brings a great value.

To test whether our approach could be a valuable tool for the identification of specific electrophysiological alterations and circadian modulations, in a second experiment, we analyzed EEG/EMG recordings coming from 14 male mice:

- a circadian mouse mutant strain: *Zfhx3*^*Sci/+*^ (*n* = 7 subjects)
- and its wild-type littermate control group: *Zfhx3*^*+/+*^ (*n* = 7 subjects).

All *Zfhx3*^*Sci/+*^ and *Zfhx3*^*+/+*^ mice were genotyped as described in [17]. For more details regarding animals’ breeding and maintenance, please refer to [13].

### Animal husbandry and data acquisition

Mice (10–14 weeks of age) were anesthetized with Ketamine/Xylazine (65/5 mg/kg, 2 ml/kg, IP) and implanted with a subcutaneous transmitter that records EEG and EMG with 2 biopotential channels (Data Sciences, F20-EET, Gold system). Electrodes (1 mm diameter) were placed on the parietal cortex of the mouse, only on 1 hemisphere. EEG electrodes were implanted on the dura, and EMG electrodes were attached to the muscle in the nape of the neck to acquire signals (Dataquest A.R.T. software, DSI) while the animals freely move in their home cage. EEG activity was sampled at 500 Hz with a 50 Hz cut-off filter. EEG signals were band-pass filtered at 0.3 Hz (low-pass filter) and 0.1 kHz (high-pass filter). After 10 days of postsurgery recovery, continuous electrophysiological signals were recorded for 24 hours (first experiment—light/dark cycle 12:12 with light switched off and on at 7 PM and 7 AM, respectively) and for 72 hours (second and third experiment—same light/dark condition with 6-hour sleep deprivation after the first 48 hours). Sleep deprivation was performed by introducing novel objects in the home cage of the animal and/or by gentle touch. All data was analyzed in 4-second epochs (the total number of epochs per subject was 21,600). The range 0.25–50 Hz was partitioned into 5 frequency bands: Delta (0.25–5 Hz), Theta (5–9 Hz), Alpha (9–12 Hz), Beta (12–20 Hz), and Gamma (20–50 Hz). Each epoch was subject to a fast Fourier transformation (FFT) with 0.48-Hz resolution using the Hanning window method. The EMG integral was exported as a measure of the muscular activity. Semiautomatic sleep scoring to annotate the 3 sleep stages (wakefulness, NREM sleep, REM sleep) was performed using the SleepSign software followed by visual inspection and correction by experts. This hand labeling was used as ground truth to better characterize our experiments. Epochs associated with artefacts (i.e., 6.74% of the dataset in the first experiment, 0.835% in the second experiment—first 24 hours of baseline; 0.655% in the third experiment—second 24 hours of baseline plus 18 hours of recovery) were excluded from the analysis.

The resulting visual scoring statistics in terms of the 3 sleep stages were as follows:

- Experiment 1: wakefulness = 56.3%, NREM sleep = 38.09%, REM sleep = 5.61%;
- Experiment 2: wakefulness = 57.17%, NREM sleep = 37.77%, REM sleep = 5.06%;
- Experiment 3 (i.e., 24 hours of baseline plus 18 hours of recovery after 6 hours of sleep deprivation): wakefulness = 54.3%, NREM sleep = 40.38%, REM sleep = 5.32%.

### Data preprocessing

A common issue to address in multisubject analysis is the variability across subjects’ physiology, which is reflected in different ranges of EEG/EMG signal for different subjects. This issue can be solved with a proper preprocessing of data. In our case, each individual dataset was preprocessed separately by first computing all possible ratios between the 5 EEG bands, because we observed that using the ratios instead of bands’ power produces less variability across subjects. A full representation of the EEG spectra is plotted in S6 Fig, showing the classical band distribution across NREM/REM stages, as determined by the manual scoring. Similarly, S7 Fig shows examples of the power bands of some latent states compared to those of the known sleep stages they are associated with. From this comparison, it can be observed that the latent states show a more compact representation of specific structure in the data than the standard sleep stages; hence, they are more specific concerning the microstructure that can be found in the data. Since our data are log-Gaussian, to deal with the skewness in the features’ distributions, we used the natural logarithm, obtaining more bell-shaped distributions [18] that facilitate the modeling process performed by the mcRBM. The zero-mean normalization was further applied to each single feature to make all the subjects comparable.

### Modeling

**Fig 8:**
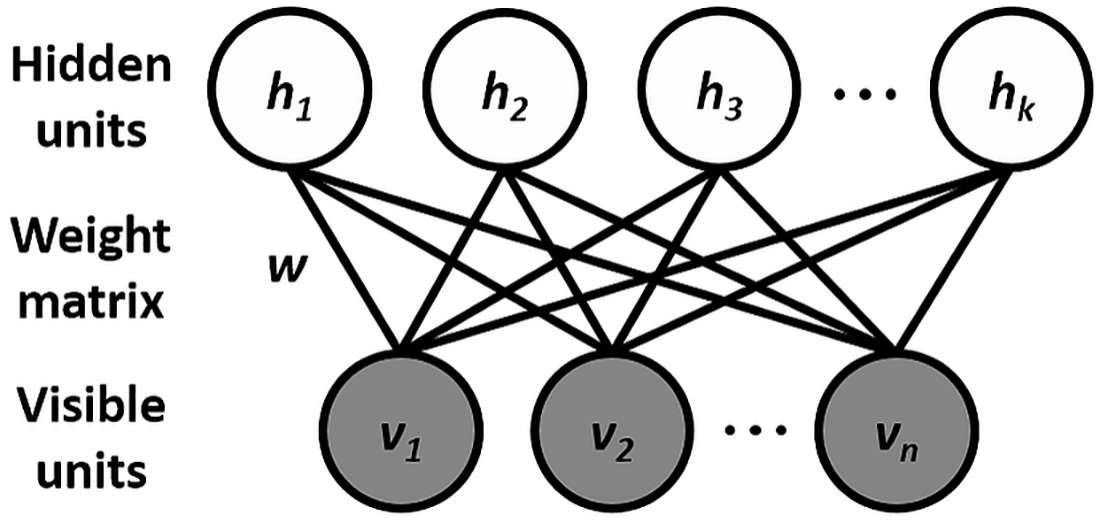
Graphical model associated to the RBM. The visible variables are represented by *v* (in our case, the ratios between the EEG frequency bands and the EMG), *h* are the latent variables, and *w* are the weights on the undirected connections between visible and hidden units. EEG, electroencephalography; EMG, electromyography; RBM, restricted Boltzmann machine.

We seek for modes in the input data distribution by modeling the joint distribution of the input variables (ratios between EEG bands and EMG) using the mcRBM [8]. The mcRBM is a probabilistic energy-based graphical model consisting of 2 fully connected layers of stochastic random variables (also called units): a layer of visible variables representing the observed data (visible units, *v*) and a layer of latent variables (hidden units, *h*) that capture dependencies between the visible ones (see Fig 8 for an illustration).

The advantage of using the mcRBM for this task is related to its effectiveness in modeling real-valued Gaussian distributed data, together with the ability to exploit the correlations between the input variables. Another important feature making the selected model more suitable than other variants of restricted Boltzmann machines (RBMs) is the fact that its latent variables are divided into 2 different sets (see section mcRBM for more details regarding the model). One of these sets ({*h*_1_, …, *h*_*m*_}) is used to model the mean value of the input variables, while the other one ({*h*_*m*+1_, …, *h*_*m+c*_}) is thought to be for explicitly modeling their covariance. This second set of latent variables allows for a better fitting of the data distribution presenting dependencies between input variables than what can be achieved with simpler models (e.g., Gaussian RBMs).

Once the model is trained, its bipartite graph structure allows for representing regularities in the observed input variables through the inferred values of the latent binary variables. In other words, the model can be used to generate a set of latent representations (the states of the inferred hidden variables) for the observed EEG/EMG data. Indeed, the model processes each input vector (corresponding to one sample of the dataset—in our case, one 4-second epoch), generating its related latent representation consisting of a series of binary values according to the probability of each single latent variable to be active.

Notably, in this Bayesian framework, the observed variables are approximately jointly Gaussian distributed [8], given the latent ones, with the mean and covariance jointly defined by the specific values of latent variables and model parameters (see Eqs 6 and 7). This means that each single latent configuration is associated with one mode in the joint distribution of the input variables, reflecting the regularities in the data. Hence, one latent representation may be considered a model representation of a sleep or a wakefulness substage.

In each of the 2 experiments performed in this study, a single mcRBM was used to model the joint distribution of the EEG ratios and the EMG. After training the model, the latent states of the 2 sets of hidden units were inferred according to Eqs 4 and 5.

The size of the mcRBM (the number of its latent variables) was set after a series of validation trials in which we “calibrated” the model to the problem at hand. We observed that having too few hidden units results in a very small number of fuzzy clusters, while, as expected, the size escalation leads to a higher computational cost. We also realized that there is a certain number of hidden units, beyond which there is no real advantage in increasing the network size, since they remain inactive. We reached a good tradeoff between computational cost and quality of the results (i.e., significantly high MI, easy interpretability of visible data distributions, good performance in groups’ discrimination, etc.) using 11 hidden covariance units (*h*_*c*_), 11 visible-to-hidden covariance factors, and 10 hidden mean units (*h*_*m*_). Similarly, all the other parameters were set after a series of trials, taking into account the average quality of the results. Finally, the models were trained on GPU over minibatches of 256 data points with learning rate set to 10^−2^.

### mcRBM

**Fig 9:**
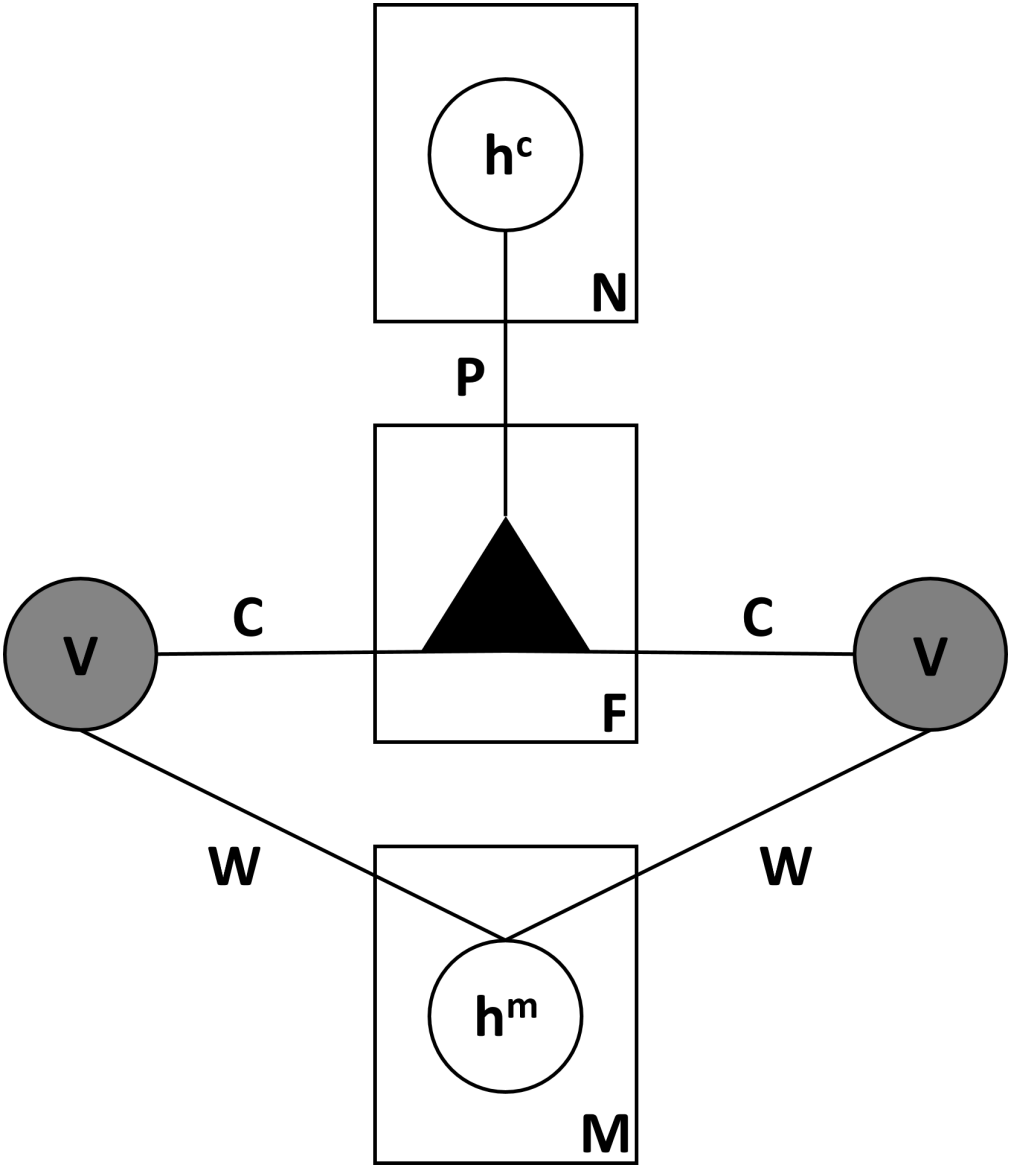
Graphical model associated with the mcRBM. *v* represents the visible variables (in our case, the EEG frequency bands and the EMG), *h*^*m*^ the latent variables modeling the mean of variables *v*, and *h*^*c*^ those modeling their covariance. *F* is the number of factors. *W* is the 2D matrix of the weights on the undirected connections between *v* and *h*^*m*^ variables. *C* is the weight matrix of the connections from the *v* variables to the factors (*F*). *P* is the weight matrix of the connections from the factors (*F*) to the *h*_*c*_. EEG, electroencephalography; EMG, electromyography; mcRBM, mean-covariance restricted Boltzmann machine.

Like all RBM models, the mcRBM [8] is a probabilistic energy-based graphical model with a bipartite undirected graph structure, which consists of 2 fully connected layers of stochastic random variables (also called units): a layer of visible variables representing the observed data (visible units, *v*), and a layer of latent variables (hidden units, *h*) that capture dependencies between the visible ones. Unlike the standard RBM models, the mcRBM has 2 groups of hidden units: mean units (*h*_*m*_) that model the mean of the input elements and precision units (*h*_*c*_) that represent pairwise dependencies between the visible variables, modeling their covariance structure (see Fig 9 for an illustration).

Due to the absence of connections between the variables within the layers, the variables of a layer are independent of each other, given the variables of the other layer. The model is defined in terms of an energy function that is given by the sum of the energy functions of the 2 groups of variables:

$$E_{mc}(v,h_{c},h_{m})=E_{c}(v,h_{c})+E_{m}(v,h_{m}).$$ (1)

The energy function of the precision variables *E*_*c*_ defines a zero-mean Gaussian distribution over the visible variables, while adding the second energy term *E*_*m*_ allows the mcRBM to produce conditional distributions over the visible units having nonzero means.

*E*_*c*_ is defined as follows:

$$E_{c}(v,h_{c})=-d^{T}h_{c}-\;(v^{T}C{)}^{2}Ph_{c},$$ (2)

in which *C* is the weight matrix from the visible (*v*) units to the factors (*F*), *P* is a weight matrix with nonpositive entries for connections from the factors (*F*) to the hidden covariance units (*h*_*c*_), and *d* is the *h*_*c*_ bias vector. *P* is constrained to have nonpositive entries in order to avoid having a model that assigns larger and larger probabilities (more negative energies) to larger and larger inputs. To control great disparity in the input features and make the model more robust, we used the normalized version of Eq 2 [8].

The energy function of mean variables *E*_*m*_ is given by:

$$E_{m}(v,h_{m})=-\frac{1}{2}(v\;-\;b{)}^{T}(v\;-\;b)c^{T}h_{m}-v^{T}Wh_{m},$$ (3)

with *W* denoting the direct connections from the hidden mean units (*h*_*m*_) to the visible ones (*v*), *b* being the visible bias, and *c* being the hidden mean bias.

After the training process, the model allows us to infer the states of its latent variables given the observed data. Given a training sample, the binary states of the *h*_*c*_ units can be inferred according to the following conditional distribution:

$$p(h_{c}|v)=\sigma (d+((v^{T}C{)}^{2}P{)}^{T}),$$ (4)

in which σ(x) = 1 / (1 + exp(-x)) is the logistic function, while the states of *h*_*m*_ units can be inferred according to the following conditional distribution:

$$p(h_{m}|v)=\sigma (c+Wv^{T}).$$ (5)

Conditioning on the latent variables, the resulting distribution over the visible ones is approximately Gaussian, which depends on both the hidden covariance and hidden mean latent states and is given by:

$$p(v|h_{c},h_{m})\;\propto \;N(\Sigma Wh_{m},\Sigma ),$$ (6)

in which *Σ* is given by:

$$\Sigma ={\left(C(diag(-P^{T}h_{c}))C^{T}\right)}^{-1}.$$ (7)

### Entropy and MI

To evaluate how well the model fits the observed data in our experiments, we analyzed how informative the learnt latent states are about the stages of wakefulness, NREM sleep, and REM sleep, as defined by the manual scoring. Since each latent state corresponds to a discrete probability distribution over the 3 manually scored stages, the informativeness we want to measure can be expressed in terms of entropy and MI.

Entropy [14] is a measure of the uncertainty of a random variable and is defined by:

$$H_{L}(Y)=-\sum_{y\in Y}p_{L}(y){log}_{2}p_{L}(y),$$ (8)

in which Y corresponds to the set of the known 3 sleep stages (wakefulness, NREM sleep, and REM sleep), while p_L_(y) is the probability of each of these 3 stages to appear in each latent state (hopefully, for each latent state, only one of the sleep stages will have a high probability).

MI [14] is a measure of the amount of information that one random variable contains about another random variable. In our case, we would like to see how informative the latent states X are about the 3 sleep stages Y. The MI I(X;Y) is the relative entropy between the joint distribution and the product distribution p(x)p(y):

$$I(X;Y)=-\sum_{x,y}p(x,y){log}_{2}\frac{p(x,y)}{p(x)p(y)},$$ (9)

in which p(x,y) is the joint probability of latent state x and sleep stage y, p(x) is the probability of latent state x, and p(y) is the probability of sleep stage y.

Finally, the NMI is computed as follows:

$$I_{n}=\frac{I(X;Y)}{H(X)}.$$ (10)

NMI (I_n_) is expressed as a real number in the range [0, 1], allowing us to compare it to its theoretical upper bound (i.e., which is equal to 1 when there is a perfect correlation between the 2 variables).

### Transition probabilities graph

Transitions between sleep stages can provide additional information regarding the structure of sleep in 24 hours. For this reason, we analyzed the transition probabilities between the observed latent states, which were computed and summarized in a squared transition probabilities matrix. The matrix was visualized as a graph whose nodes are the latent states and edges are the transitions between them. The weight of each edge is the probability of the corresponding transition. We used the ForceAtlas2 [15] algorithm to cluster the network. Briefly, the algorithm randomly positions the nodes in a 2D space, which are then moved according to the attraction and repulsion forces among the nodes until the network reaches a state of equilibrium. These forces cause nodes with a big number of connections (hubs) to strongly repel each other and nodes connected with a heavily weighted edge (meaning a very probable transition) to attract each other [15].

### Group discrimination

One question we addressed was whether we could use the discovered latent states to discriminate between different mouse genotypes. To do so, each subject was represented with a discrete probability distribution over the k = 1,2, …, *n* observed latent states, with P_i_(x_k_) being the probability of latent state k for subject i.

We then performed a classification with a leave-one-subject-out schema (train the model on *N* − 1 subjects and test over the left-out subject, iterating *N* times over all *N* subjects). We used an ensemble of classifiers based on a linear support vector machine (SVM), a linear discriminant analysis (LDA), and a 1-nearest neighbor (1-NN) to classify with a majority vote the left-out subject as belonging to 1 of the 3 groups.

## Supporting information

S1 Fig Examples of input data (bands’ ratios) distributions associated with 3 latent states corresponding with high probability to (A) REM sleep state and (B) wakefulness state. Top: distributions of bands’ ratios along the input samples associated with the represented latent state are visualized in a box plot. Bottom: matrices describe for each latent state the pairwise covariance between all input variables. REM, rapid-eye-movement.
(TIF)

S2 Fig Strains’ discrimination results with an ensemble classifier composed by an LDA, a linear SVM, and a 1-NN classifier. Confusion matrix showing the detailed misclassification errors, in which we can see that only 2 subjects for each strain have been misclassified. Interestingly, we can also observe that both misclassified subjects of the mixed background strain have been classified as belonging to the C57BL/6J group. This can be due to the fact that C57BL/6J background and mixed background groups are genetically related. 1-NN, 1-nearest neighbor; LDA, linear discrimination analysis; SVM, support vector machine.
(TIF)

S3 Fig Transition probabilities graphs of the 3 wild-type mouse models: (A) C57BL/6J; (B) CD1; (C) mixed background. Nodes correspond to latent states, edges to transitions between them. Nodes’ size is related to their in-degree. Blue, green, and red nodes are associated with latent states mapping with high probability to wakefulness, NREM sleep, and REM sleep, respectively. Purple nodes correspond to substages that cannot be clearly associated to any of the known sleep states. Edges are weighted with the probability of the corresponding transition and colored according to the source node. Graphs were built using the ForceAtlas2 algorithm [15,16]. See also the interactive graphs available at http://pavis.iit.it/datasets/mouse-sleep-analysis. NREM, non-rapid eye movement; REM, rapid eye movement.
(TIF)

S4 Fig Daily profiles. Examples of circadian patterns associated with (A) wakefulness, (B) NREM sleep, and (C) REM sleep. NREM, non-rapid eye movement; REM, rapid eye movement.
(TIF)

S5 Fig Daily profiles of the 3 sleep stages according to the manual scoring. (TIF)

S6 Fig Box plots associated to bands’ power, representing the full EEG spectra for the 3 groups of wild-type mice (C57BL/6J, CD1, mixed background) in NREM and REM sleep. EEG, electroencephalography; NREM, non-rapid eye movement; REM, rapid eye movement.
(TIF)

S7 Fig Left: box plots of the bands’ power of the latent states in Fig 3 that are characteristic of the 3 genetic backgrounds (A—C57BL/6J, B—CD1, C—mixed background) compared to the box plots of the power of the known according to the GT stage (right) they are associated with. GT, ground truth.
(TIF)

S1 Data Data associated with the statistics summarized in the bar plots in Fig 5. (XLSX)

S2 Data Data associated with the statistics summarized in the bar plots in Fig 7A. (XLSX)

S1 Checklist ARRIVE checklist. (PDF)

## Abbreviations

1-NN: 1-nearest neighbor
EEG: electroencephalography
EMG: electromyography
FFT: fast Fourier transformation
LDA: linear discriminant analysis
mcRBM: mean-covariance restricted Boltzmann machine
MI: mutual information
NMI: normalized mutual information
NREM: non-rapid eye movement
RBM: restricted Boltzmann machine
REM: rapid eye movement
SVM: support vector machine
ZT: zeitgeber time
