# Supplementary figures and images for: A novel unsupervised analysis of electrophysiological signals reveals new sleep substages in mice
Source: PLoS Biol. 2018 May 29;16(5):e2003663. doi: 10.1371/journal.pbio.2003663 (PMC5993302; doi:10.1371/journal.pbio.2003663)

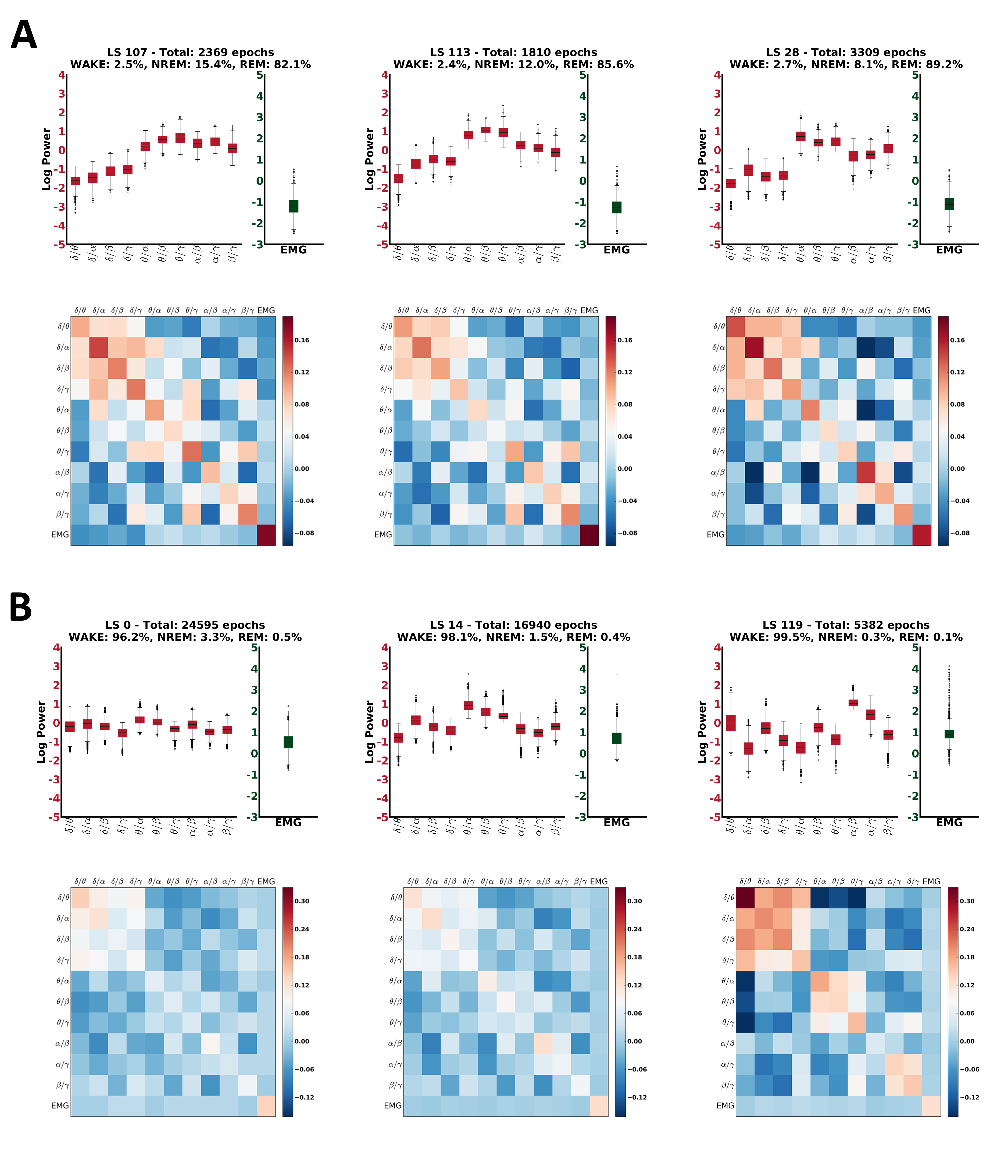

Supplement: S1 Fig — Top: distributions of bands’ ratios along the input samples associated with the represented latent state are visualized in a box plot. Bottom: matrices describe for each latent state the pairwise covariance between all input variables. REM, rapid-eye-movement. (TIF) [file pbio.2003663.s001.tif]

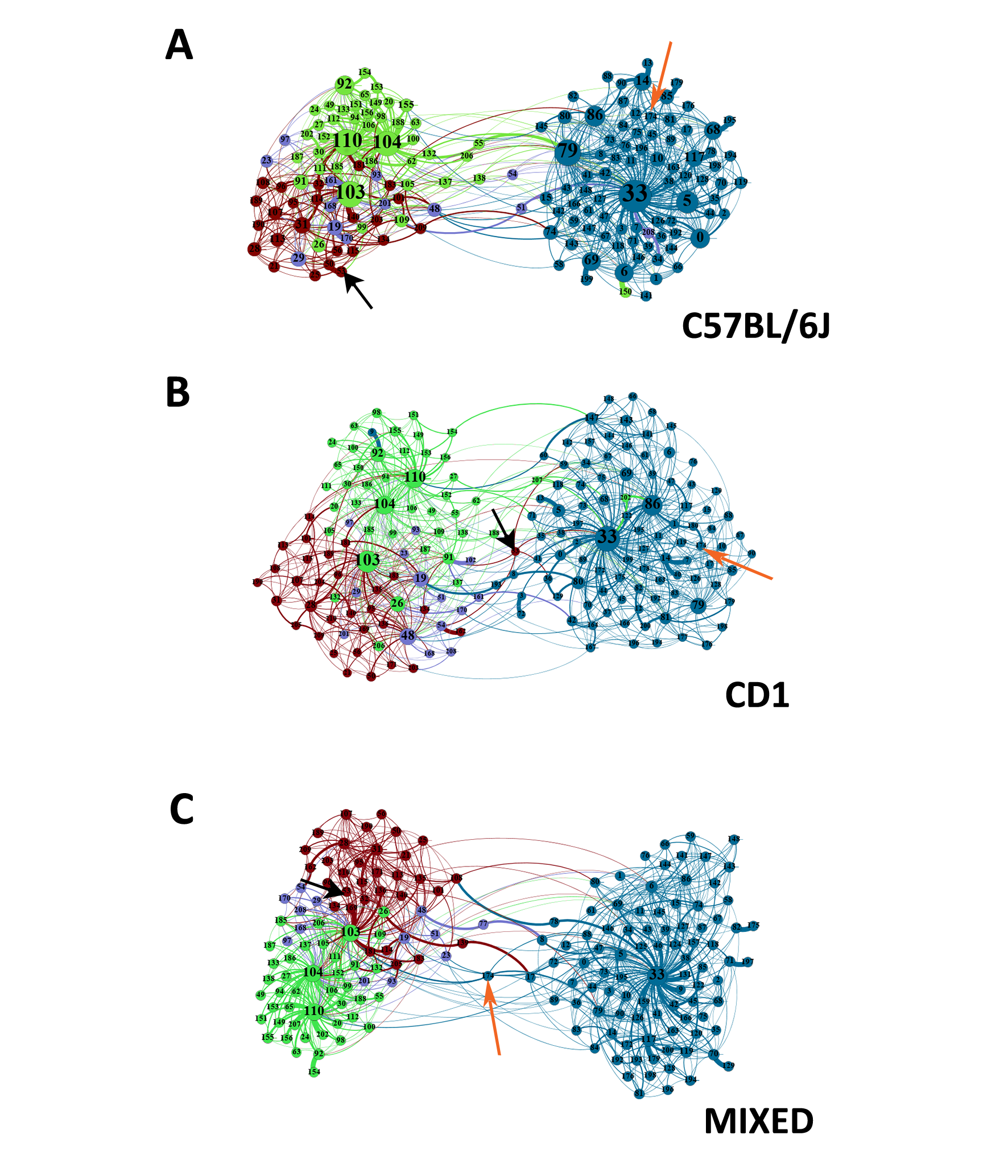

Supplement: S3 Fig — Nodes correspond to latent states, edges to transitions between them. Nodes’ size is related to their in-degree. Blue, green, and red nodes are associated with latent states mapping with high probability to wakefulness, NREM sleep, and REM sleep, respectively. Purple nodes correspond to substages that cannot be clearly associated to any of the known sleep states. Edges are weighted with the probability of the corresponding transition and colored according to the source node. Graphs were built using the ForceAtlas2 algorithm [15,16]. See also the interactive graphs available at http://pavis.iit.it/datasets/mouse-sleep-analysis. NREM, non-rapid eye movement; REM, rapid eye movement. (TIF) [file pbio.2003663.s003.tif]

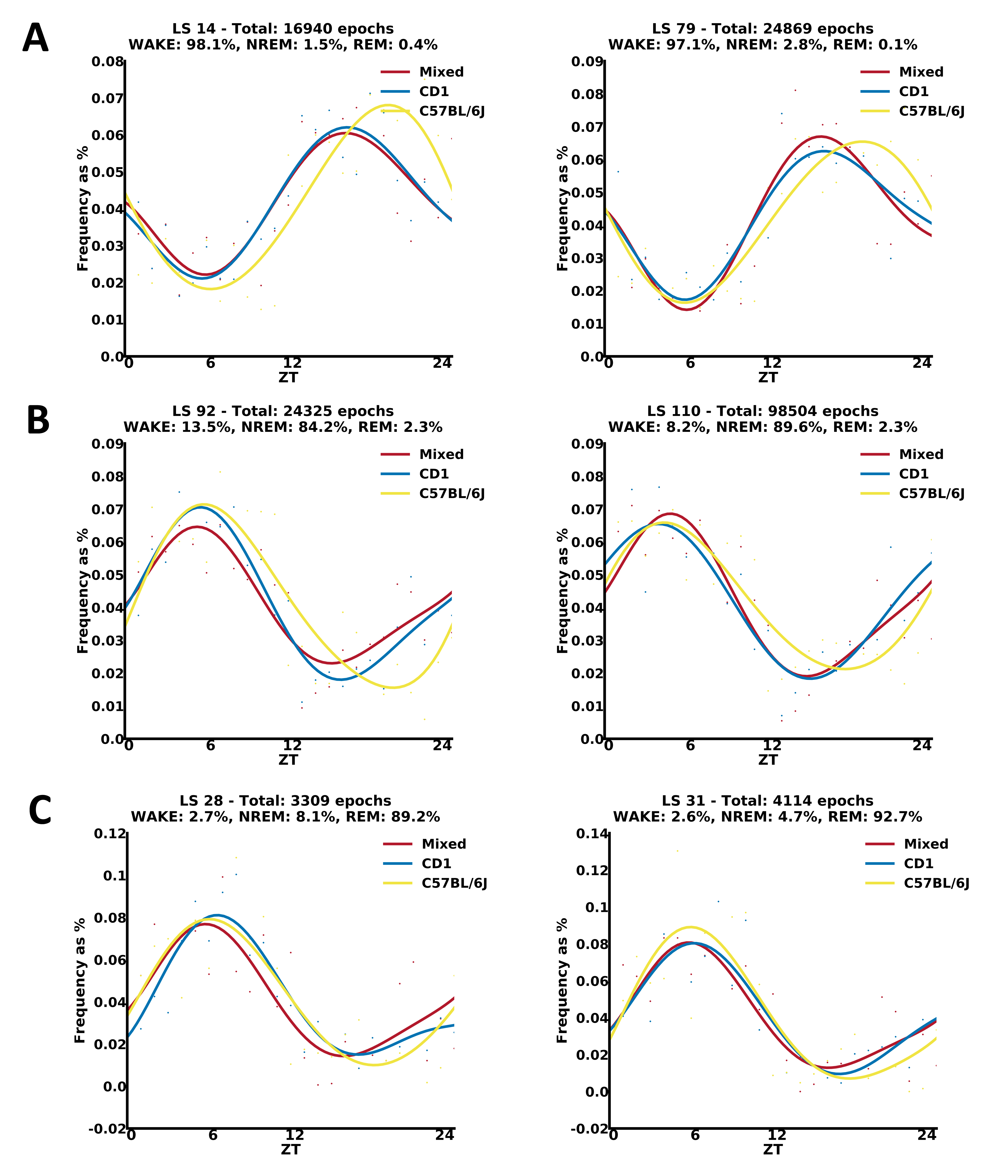

Supplement: S4 Fig — Examples of circadian patterns associated with (A) wakefulness, (B) NREM sleep, and (C) REM sleep. NREM, non-rapid eye movement; REM, rapid eye movement. (TIF) [file pbio.2003663.s004.tif]

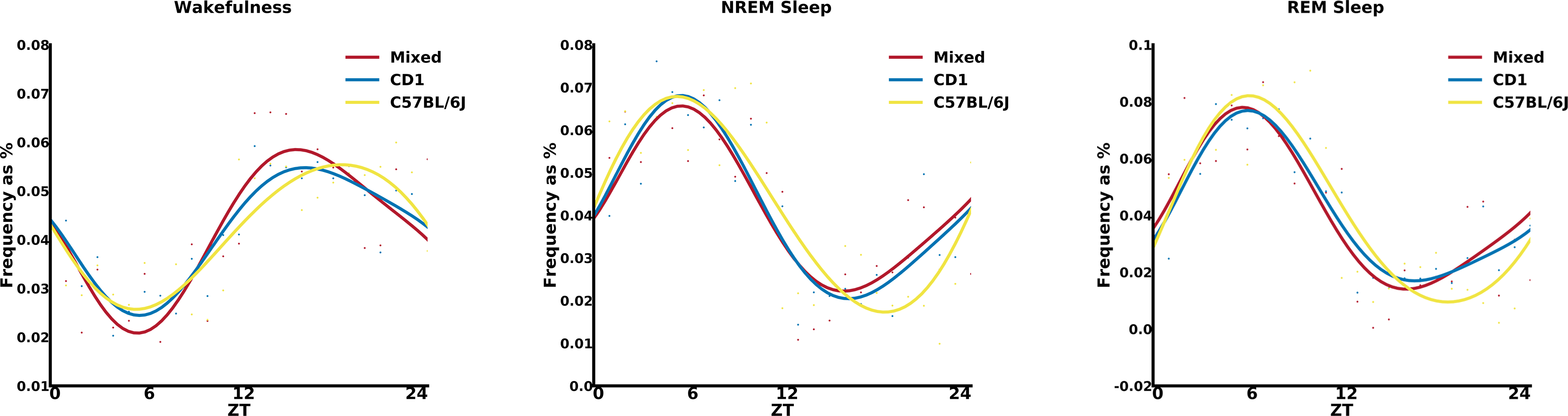

Supplement: S5 Fig — (TIF) [file pbio.2003663.s005.tif]

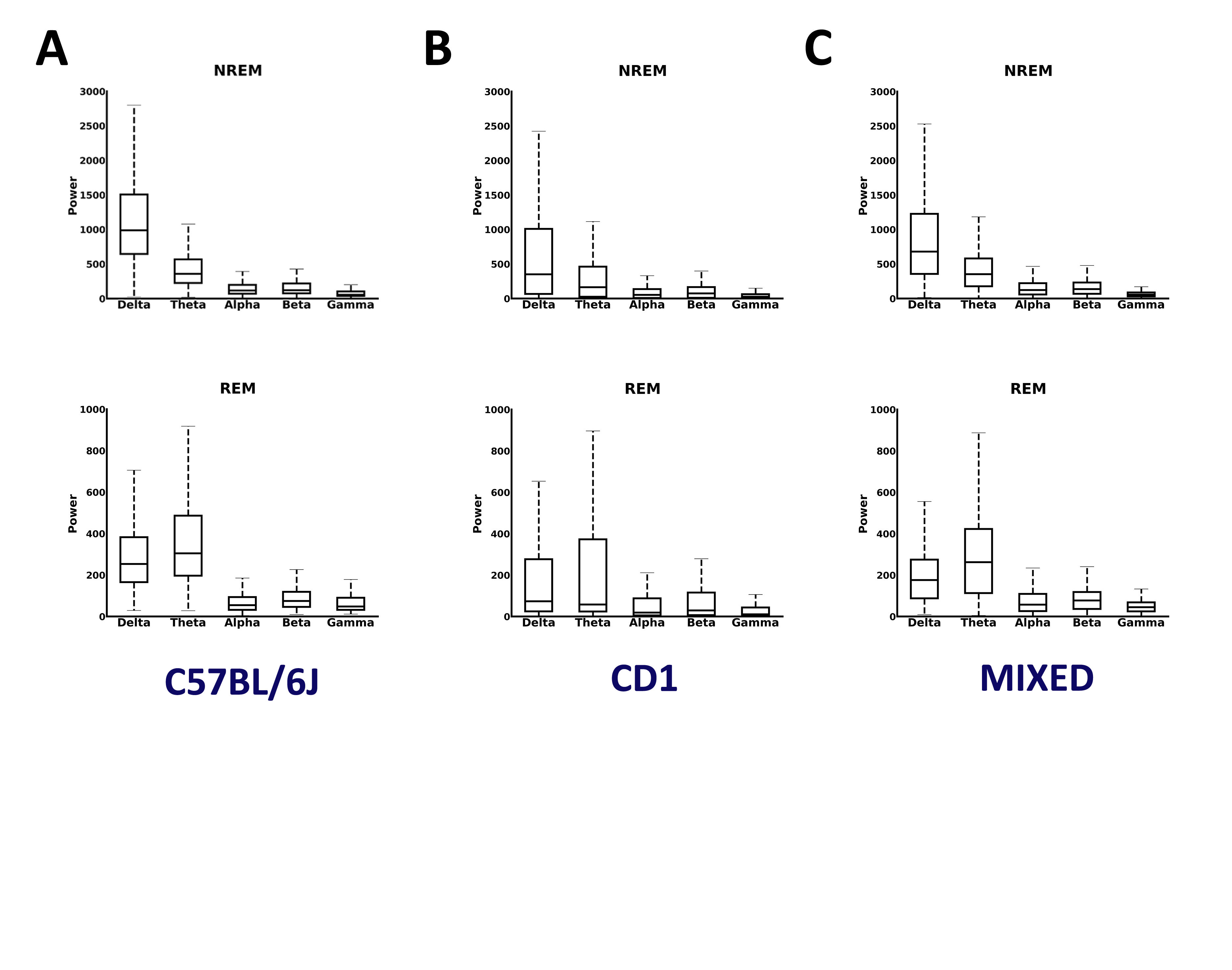

Supplement: S6 Fig — EEG, electroencephalography; NREM, non-rapid eye movement; REM, rapid eye movement. (TIF) [file pbio.2003663.s006.tif]

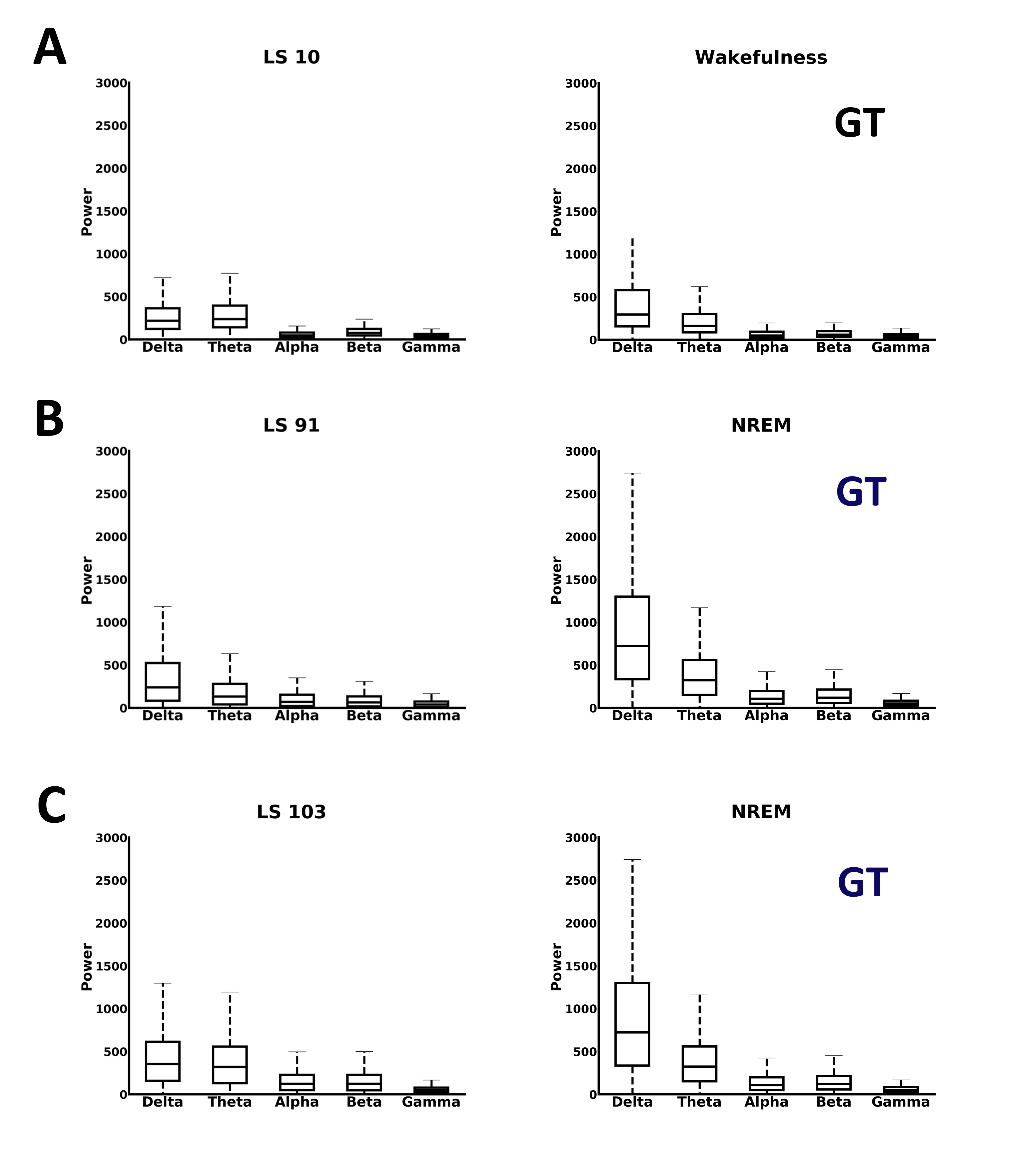

Supplement: S7 Fig — GT, ground truth. (TIF) [file pbio.2003663.s007.tif]
